# Dietary Patterns and Weight Status of Primary School Children in Serbia

**DOI:** 10.3389/fpubh.2021.678346

**Published:** 2021-06-15

**Authors:** Predrag Bozic, Visnja Djordjic, Lidija Markovic, Dragan Cvejic, Nebojsa Trajkovic, Sabolc Halasi, Sergej Ostojic

**Affiliations:** ^1^Serbian Institute of Sport and Sports Medicine, Belgrade, Serbia; ^2^Faculty of Sport and Physical Education, University of Montenegro, Niksic, Montenegro; ^3^Faculty of Sport and Physical Education, University of Novi Sad, Novi Sad, Serbia; ^4^Faculty of Education, University of Novi Sad, Sombor, Serbia; ^5^Faculty of Sport and Physical Education, University of Nis, Nis, Serbia; ^6^Hungarian Language Teacher Training Faculty, University of Novi Sad, Subotica, Serbia

**Keywords:** obesity, overweight, underweight, WHO childhood obesity surveillance initiative, food habits, breakfast

## Abstract

The purpose of the present cross-sectional study was to examine dietary patterns and the prevalence of underweight, overweight, and obesity among Serbian children. Furthermore, the study analyzed the association between dietary patterns and weight status. A nationally representative sample of 6–9-year-old children (*n* = 3,067) was evaluated as part of the Fifth Round World Health Organization European Childhood Obesity Surveillance Initiative. The children's height and weight were measured by trained field examiners, while their parents or guardians filled paper versions of the food frequency questionnaire to collect information related to the child's breakfast habits and food and beverage intake. According to the International Obesity Task Force cut-off points, the overall prevalence of overweight (including obesity) and underweight were 28.9 and 8.1%, respectively. The majority of parents reported that their children (84.5%) had breakfast every day, while only 39.5 and 37% of children had daily fruit and vegetable consumption, respectively. The children who do not eat breakfast every day are more likely to be obese (OR = 1.50), while a higher intake frequency of nutrient-poor beverages such as soft drinks increases the risk of being not only overweight (OR = 1.32) but also underweight (OR = 1.39). Regular monitoring and understanding of dietary patterns and weight status is crucial to inform, design, and implement strategies to reduce national and global diet and obesity-related diseases. Urgent actions need to be taken from public policymakers to stop and reverse the increasing trend of overweight (including obesity) among Serbian children.

## Introduction

Overweight and obesity in children are among the most severe public health problems that have increased dramatically during the last decades at the global and European levels (1). According to estimates from the World Health Organization (WHO), around 1 in 3 children in Europe aged 6–9 years old were overweight or obese (2), while reports from eight European countries suggest varied prevalence for underweight, from 5.7% in Italian boys to 16.6% for girls in Hungary (3, 4). Being overweight during childhood is associated with obesity and adverse health consequences throughout the life-span (5), reducing the average age at which non-communicable diseases and disabilities or the likelihood of mortality become apparent (6). Although it has received less scientific scrutiny and is not regularly monitored, thinness or underweight could also harmfully affect child health, growth, and well-being in various ways, including nutritional deficiencies, menstrual irregularity, impaired immune system, osteoporosis, anemia, anorexia nervosa, hypotension, and decreased cognitive and work capacity (7–10).

Among all the behavioral factors, dietary patterns in childhood are considered one of the most important contributors to childhood malnutrition (stunting, wasting, micronutrient deficiencies, obesity) (11–13) and non-communicable diseases (14). Low-quality diets are believed to be the biggest and modifiable behavioral risk factors for the global burden and disease (15). The average dietary patterns among children and adolescents are shifted to more prepared and processed fast-foods and diets high in fat, sugar, and salt (16, 17). Unhealthy habits, such as skipping breakfast (18) and eating outside the home more frequently (1), increase the consumption of high energy-dense and nutrition poor foods. It further decreases the intake of key food groups such as fruit, vegetable, dairy, and dietary fiber and reduces the likelihood of meeting the recommendations for micronutrients (19). Dietary habits acquired during childhood tend to persist into adulthood (20), making nutrition in childhood an essential public health issue.

Regular monitoring of weight status and dietary habits would ideally be part of a nationally representative surveillance system (21) that could provide comparable data among children necessary for evaluating the effectiveness of obesity prevention efforts (22). As a response to the need for a European-wide harmonized surveillance system, WHO Regional Office for Europe initiated in 2006 the Childhood Obesity Surveillance Initiative (COSI) for children aged 6–9 years. Over 40 European countries participated in the five rounds of COSI so far. However, many of them provided reports only on mandatory measurements such as the prevalence of overweight and obesity, not including data on lifestyle behaviors such as food consumption frequency (23). Serbia joined COSI in the fourth round and reported an overall prevalence of overweight (including obesity) and underweight of 23.1 and 9.6%, respectively, in data collected from a nationally representative sample of 6–9-year-old school children (3, 24). However, regular reports on the weight status and dietary patterns of Serbia's schoolchildren are lacking. In addition, although dietary factors have been considered as one of the most important contributors to childhood obesity, overweight and underweight, this relationship is complex and not fully understood (25). Therefore, research on the issues of factors associated with overweight and underweight in schoolchildren deserves further attention.

Given the importance of nutrition and weight status in childhood, the present study aimed to explore (i) the prevalence of underweight, overweight, and obesity in a nationally representative sample of 6–9-year-old Serbian children, (ii) the parental reports of frequency of breakfast, selected food, and beverages intake, as well as (iii) investigate whether there is an association between dietary patterns and weight status.

## Methods

Following the COSI protocol (26), a cross-sectional design was employed in the present study selecting primary school children aged 6–9.9 years (Table 1). A nationally representative sample was selected through cluster sampling, with the primary school as the primary sampling unit. The minimal planned sample size consisted of 2,400 children as recommended by the WHO European Office. Primary schools were selected randomly from the list of public primary schools provided by the Serbian Ministry of Education. Since < 1% of the target children were enrolled in private or special schools, these schools were excluded from the sampling frame. The selection process employed proportional sampling procedures based on NUTS levels 2 and 3 (statistical regions and districts) and level of urbanization. Two criteria were used to ascribe a settlement to an urban/rural category: (1) administrative classification and (2) population size. Eventually, three categories were defined: (1) urban—more than 100,000 inhabitants, (2) semiurban—up to 100,000 inhabitants, and categorized as towns by administrative classification; and (3) rural—small towns and villages, not categorized as towns by administrative classification. All children registered in the sampled classes were invited to take part in the study. Those children who returned a signed parental consent and accepted to participate in the study on the survey day were examined and received the food frequency questionnaire. All data were collected during the spring of 2019 as a part of COSI round five. The study is conducted in accordance with the Declaration of Helsinki, and all procedures were approved by the World Health Organization (No. 2018/873491-0). Parents, teachers, and school administrators were fully informed about all study procedures, while signed informed consent for the measurements and data treatment was obtained voluntarily before the child's enrollment in the study. Verbal consent from the child to participate in the study was obtained on the measurement day.

**Table 1:** Anthropometric characteristics with prevalence of thinness, normal weight, overweight, and obesity for a representative sample of Serbian children.

	**Total mean (SD)**	**Girls mean (SD)**	**Boys mean (SD)**
**General information**	***n =*** **3,067**	***n =*** **1,440**	***n =*** **1,627**
Age (years)	8.5 (0.8)	8.5 (0.8)	8.5 (0.8)
Height (cm)	134.0 (7.8)	133.6 (7.8)^**^	134.4 (7.7)
Weight (kg)	31.7 (7.9)	31.3 (7.7)^*^	32.0 (8.0)
BMI (kg/m^2^)	17.4 (3.0)	17.3 (3.0)	17.5 (3.0)
**Body weight status (IOTF)**	***n*** **(%)**	***n*** **(%)**	***n*** **(%)**
Thinness	247 (8.1)	125 (8.7)	122 (7.5)
Normal	1,935 (63.1)	898 (62.7)	1,037 (63.7)
Owerweight	635 (20.7)	287 (19.9)	348 (21.4)
Obesity	250 (8.2)	130 (9.0)	120 (7.4)

* *p < 0.05*,

** *p < 0.01*.

During the data collection period, 3,397 children from 57 public elementary schools were recruited. Only two schools refused to participate and 55 schools from all 5 statistical regions (NUTS level 2) in Serbia and 26 out of 29 districts (NUTS level 3) participated in the study. After initial checking, 162 children were excluded due to missing or inaccurate data and 168 due to age outside the limits acceptable for the study (6–9.99). The final sample consisted of 3,067 children (47% girls). The final sample consisted of 68.3% urban/semiurban schools and 32.7% rural schools.

The height and weight of the children were measured by five trained field examiners (physical education teachers). Children were measured in everyday clothes without their shoes and heavy objects like wallets, mobile phones, key chains, belts, and hair ornaments. Body weight was measured to the nearest 0.1 kg with portable digital scales (Omron BF214, Kyoto, Japan) and was corrected for the average weight of the clothes worn (gym clothes −0.15 kg, light clothing −0.35 kg, heavy clothing −0.5 kg). Body height was measured to the nearest 0.1 cm using a portable stadiometer (Seca 213, Hamburg, Germany). Age and gender-specific body mass index (BMI, kg/m^2^) cut-off points for different categories of body weight status (thinness, normal weight, overweight, and obesity) were applied according to widely used classification, the International Obesity Task Force (IOTF) (27).

The food frequency questionnaire was used to collect information related to the child's breakfast habits and food and beverage intake. The food frequency items are part of the Family Record Form, a questionnaire designed within the WHO COSI project and widely used in many international studies (28). The food frequency items had been adapted from the 2001/2002 questionnaire of the Health Behavior in School-aged Children study (HBSC study) validated on adolescent samples (29). The development of the national version followed the COSI Protocol and the original form (food frequency items included) was translated into the Serbian language and back-translated into English by a professional translator. To provide further information on the clarity and validity of the questionnaire, it was pilot tested on a small sample of the parents (*n* = 40). The meaning and clarity of the items were discussed with the pilot study participants after completing the questionnaires. The principal investigator approved the final version of the questionnaire (the questionnaire is available upon the request to the corresponding author). Paper versions of the questionnaire were presented to the parents and children during the measurements or given to the children to bring home to be filled by their parents or guardians. The question relating to breakfast habits was formulated as follows: over a typical week, how often does your child have breakfast? The allowed options for answers were “never,” “some days (1–3 days),” “most days (4–6 days),” and “every day.” Seventeen items were contained in the food and beverage part of the questionnaire (Table 2), and the question was: How often does your child eat or drink the following kinds of foods or beverages? The options for food items and beverages were “never,” “less than once a week,” “some days (1–3 days),” “most days (4–6 days),” and “every day.” For the purpose of inferential statistics, the response for the breakfast intake, fruit and vegetable consumption were dichotomized to “every day” and “ <7 days a week,” while the responses for soft drink, savory snacks, and sweets were dichotomized into “ <1 days a week” and “one or more days a week.”

**Table 2:** Breakfast, food, and beverages frequency of consumption for Serbian children during a regular week.

	**Girls n (%)**					**Boys n (%)**					**Total n (%)**				
	**Never**	**Less than once a week**	**Some days (1–3 days)**	**Most days (4–6 days)**	**Every day**	**Never**	**Less than once a week**	**Some days (1–3 days)**	**Most days (4–6 days)**	**Every day**	**Never**	**Less than once a week**	**Some days (1–3 days)**	**Most days (4–6 days)**	**Every day**
Breakfast frequency	22 (1.7)		87 (6.7)	100 (7.7)	1,082 (83.8)	27 (1.9)		87 (6)	101 (7)	1,228 (85.1)	49 (1.8)		174 (6.4)	201 (7.4)	2,310 (84.5)
**Food and beverages frequancy**															
Fresh_fruit	8 (0.6)	35 (2.7)	304 (23.5)	407 (31.5)	537 (41.6)	13 (0.9)	67 (4.7)	344 (24.1)	467 (32.7)	536 (37.6)	21 (0.8)	102 (3.8)	648 (23.8)	874 (32.2)	1,073 (39.5)
Vegetables (excluding potatos)^*^	12 (0.9)	33 (2.6)	264 (20.6)	484 (37.8)	487 (38)	18 (1.3)	42 (3)	338 (23.8)	510 (35.9)	511 (36)	30 (1.1)	75 (2.8)	602 (22.3)	994 (36.8)	998 (37)
Cereals	374 (33.5)	292 (26.2)	334 (29.9)	70 (6.3)	46 (4.1)	500 (39.5)	315 (24.9)	342 (27)	61 (4.8)	48 (3.8)	874 (36.7)	607 (25.5)	676 (28.4)	131 (5.5)	94 (3.9)
Legumes	38 (3)	326 (25.7)	750 (59.1)	128 (10.1)	27 (2.1)	67 (4.7)	346 (24.5)	837 (59.2)	131 (9.3)	32 (2.3)	105 (3.9)	672 (25.1)	1,587 (59.2)	259 (9.7)	59 (2.2)
Egg dishes	14 (1.1)	127 (10)	765 (60.3)	296 (23.3)	66 (5.2)	18 (1.3)	130 (9.3)	827 (58.9)	345 (24.6)	83 (5.9)	32 (1.2)	257 (9.6)	1,592 (59.6)	641 (24)	149 (5.6)
Fish	113 (8.9)	665 (52.5)	455 (35.9)	28 (2.2)	6 (0.5)	138 (9.8)	723 (51.5)	509 (36.3)	26 (1.9)	7 (0.5)	251 (9.4)	1,388 (52)	964 (36.1)	54 (2.0)	13 (0.5)
Meat	8 (0.6)	24 (1.9)	372 (29.1)	599 (46.9)	274 (21.5)	12 (0.9)	22 (1.6)	387 (27.5)	690 (49)	298 (21.1)	20 (0.7)	46 (1.7)	759 (28.3)	1,289 (48)	572 (21.3)
Skimmed/semi- skimmed milk	244 (19.8)	162 (13.1)	281 (22.8)	253 (20.5)	292 (23.7)	314 (23.3)	156 (11.6)	290 (21.5)	252 (18.7)	338 (25)	558 (21.6)	318 (12.3)	571 (22.1)	505 (19.6)	630 (24.4)
Whole fat milk	262 (22.1)	222 (18.8)	274 (23.1)	218 (18.4)	208 (17.6)	299 (22.5)	220 (16.5)	305 (22.9)	246 (18.5)	261 (19.6)	561 (22.3)	442 (17.6)	579 (23)	464 (18.4)	469 (18.6)
Flavored milk	453 (36.9)	355 (28.9)	264 (21.5)	86 (7.0)	69 (5.6)	520 (38.1)	374 (27.4)	318 (23.3)	81 (5.9)	71 (5.2)	973 (37.6)	729 (28.1)	582 (22.5)	167 (6.4)	140 (5.4)
Cheese	112 (8.9)	252 (20.1)	490 (39)	273 (21.8)	128 (10.2)	175 (12.6)	265 (19.1)	553 (39.8)	254 (18.3)	144 (10.4)	287 (10.8)	517 (19.5)	1,043 (39.4)	527 (19.9)	272 (10.3)
Yogurt and other dairy products^a^	13 (1.0)	68 (5.3)	352 (27.6)	422 (33.1)	421 (33)	26 (1.8)	73 (5.1)	379 (26.7)	424 (29.9)	517 (36.4)	39 (1.4)	141 (5.2)	731 (27.1)	846 (31.4)	938 (34.8)
Fuit juice (100%)	84 (6.7)	306 (24.3)	425 (33.8)	261 (20.7)	183 (14.5)	83 (5.9)	311 (22.2)	497 (35.4)	297 (21.2)	214 (15.3)	167 (6.3)	617 (23.2)	922 (34.6)	558 (21)	397 (14.9)
Soft drinks that contain sugar	131 (10.4)	354 (28.1)	432 (34.3)	177 (14)	166 (13.2)	142 (10.2)	344 (24.6)	496 (35.5)	226 (16.2)	191 (13.7)	273 (10.3)	698 (26.3)	928 (34.9)	403 (15.2)	357 (13.4)
Diet drinks	941 (76.9)	157 (12.8)	79 (6.5)	20 (1.6)	26 (2.1)	1,057 (77.9)	187 (13.8)	79 (5.8)	17 (1.3)	17 (1.3)	1,998 (77.4)	344 (13.3)	158 (6.1)	37 (1.4)	43 (1.7)
Savory snacks^b^	32 (2.5)	310 (24.1)	551 (42.9)	230 (17.9)	161 (12.5)	50 (3.5)	312 (22)	634 (44.8)	247 (17.4)	173 (12.2)	82 (3.0)	622 (23)	1,185 (43.9)	477 (17.7)	334 (12.4)
Sweets^c^	13 (1.0)	119 (9.3)	521 (40.7)	362 (28.3)	264 (20.6)	15 (1.1)	122 (8.6)	587 (41.5)	410 (29)	279 (19.7)	28 (1.0)	241 (9)	1,108 (41.2)	772 (28.7)	543 (20.2)

a *Yogurt, milk pudding, cream cheese, or other dairy products*.

b *Potato crisps, corn crisps, popcorn, or peanuts*.

c *Candy bars or chocolates*.

* *Girls more frequently were reported to consume vegetables more than 4 days a week*.

Descriptive statistics, mean and standard deviation (SD), were calculated for all anthropometric variables. In addition, the frequency of breakfast and food consumption was calculated according to gender and the weight status category. The gender differences in the prevalence of child thinness, overweight, and obesity as well as in the distribution of responses obtained for breakfast and selected food and beverage consumption, were assessed using the chi-square test with Bonferroni correction (30). The same tests were also applied to identify significant independent variables between the different weight status categories and the distribution of responses obtained for breakfast and food/beverage consumption. Odds ratios (ORs) with 95% confidence intervals (CIs) were computed to estimate the association between variables. The data were analyzed using the statistical package (SPSS Statistics 21.0, IBM Corporation, Chicago, IL, USA).

## Results

The mean and SD for age, weight, height, and prevalence rate for thinness, normal weight, overweight, and obesity are presented in Table 1. We found lower values in girls for body height (*p* < 0.01) and weight (*p* < 0.05) but not for BMI or neither for distribution in the IOTF body weight status categories. The total percentage of overweight children, including obesity, was 28.9%.

Breakfast, food and beverages frequency of consumption for Serbian children during a regular week were presented in Table 2. We did not find any gender differences except for vegetable consumption (*p* < 0.05); girls more commonly were reported as having vegetables more than 4 days a week.

Differences among weight status categories (thinness, normal, overweight, obesity) and reported frequency for breakfast and food/beverages consumption are presented in Table 3. A lower frequency of consumption of savory snacks was reported in the normal group (*p* < 0.01). The risk of being obese was higher if the child was reported not to have breakfast every day [OR = 1.50 (1.05–2.14), *p* = 0.03] or have a higher intake frequency of soft drinks more than 1 day a week [OR = 1.38 (1.01–1.88), *p* = 0.04]. The risk of being overweight [OR = 1.32 (1.08–1.61), *p* = 0.01] or underweight [OR = 1.39 (1.02–1.88), *p* = 0.04] was also higher if the child was reported to have a higher intake frequency of soft drinks more than 1 day a week.

**Table 3:** Breakfast, selected food, and beverages frequency of consumption for children with different weight status.

	**Weight status**				
	**Thinness *n* (%)**	**Normal weight *n* (%)**	**Overweight *n* (%)**	**Obesity *n* (%)**	**Total *n* (%)**
**Breakfast**					
Every day	184 (82.1)	1,461 (85.2)	496 (85.2)	169 (79.3)	2,310 (84.5)
<7 days a week	40 (17.9)	254 (14.8)	86 (14.8)	44 (20.7)	424 (15.5)
**Fresh_fruit**					
Every day	82 (36.9)	695 (40.9)	217 (37.3)	79 (36.7)	1,073 (39.5)
<7 days a week	140 (63.1)	1,005 (59.1)	364 (62.7)	136 (63.3)	1,645 (60.5)
**Vegetables (excluding potatos)**					
Every day	85 (38.8)	632 (37.4)	203 (35)	78 (37)	998 (37)
<7 days a week	134 (61.2)	1,057 (62.6)	377 (65)	133 (63)	1,701 (63)
**Soft drinks**					
<1 days a week	68 (31.6)	649 (39.1)^**^	188 (32.7)	66 (31.7)	971 (36.5)
>1 days a week	147 (68.4)	1,012 (60.9)^**^	387 (67.3)	142 (68.3)	1,688 (63.5)
**Savory snacks^a^**					
<1 days a week	49 (22.1)	432 (25.6)	167 (29)	56 (26.4)	704 (26.1)
>1 days a week	173 (77.9)	1,258 (74.4)	409 (71)	156 (73.6)	1,996 (73.9)
**Sweets^b^**					
<1 days a week	19 (8.5)	182 (10.8)	45 (7.8)	23 (10.9)	269 (10)
>1 days a week	204 (91.5)	1,498 (89.2)	533 (92.2)	188 (89.1)	2,423 (90)

a *Potato crisps, corn crisps, popcorn, or peanuts*.

b *Candy bars or chocolates*.

** *p < 0.01*.

## Discussion

The present study investigated the weight status and dietary patterns of 6–9-year-old Serbian children. Our findings showed that the overall prevalence of overweight (including obesity) and underweight was 28.9 and 8.1%, respectively, without differences between genders. Regarding the dietary patterns, a high level of daily breakfast intake has been observed among Serbian children (84.5%). At the same time, consumption of some healthy food items, such as fruits and vegetables, were below the recommended level, while daily consumption of nutrient-poor foods such as savory snacks, sweets, and soft drinks was higher than the pooled estimates observed on the European level. The children who do not eat breakfast every day are more likely to be overweight and obese, while a higher intake frequency of nutrient-poor beverages such as soft drinks increases the risk of being not only overweight but also underweight.

The present study represents the second survey that employed a standardized COSI protocol to assess Serbian children's weight status. According to the IOTF cut-off points, the findings suggest the overall prevalence of overweight 20.7%, obesity 8.2%, and underweight 8.1% in the country representative sample of primary-school children. Although significant differences have been found in body height and weight, no significant gender differences were identified for BMI or prevalence in any weight status categories. The findings from the present study seem to be comparable to relatively high rates of overweight previously reported in other countries participating in the COSI program (23). Compared to other European countries, Serbia seems to fit the Central and Eastern Europe overweight/obesity profiles in children (11, 23). At the same time, the prevalence rates are generally higher compared to the rates in Western, Baltic, and Nordic regions and considerably lower than overweight ratios in Southern Europe (Spain, Cypar, Greece, Italy) (11, 23). In comparison to thinness prevalence, it seems that findings from our study do not differ from ones observed in eight European countries (4). Our previous research performed as a part of the fourth round of COSI revealed lower values of overweight and obesity (16.2 and 6.9%, respectively) (24) and slightly higher underweight values (9.6%) (3). The relatively higher overall prevalence of overweight (including obesity) observed in the fifth COSI round (28.9%) compared to the fourth round (23.1%) should be interpreted as a red alarm to trigger urgent actions focused on stopping obesity pandemic present in Serbian children.

Regarding the dietary patterns, we found some positive sides but also many areas that require improvements. A wealth of research suggests a significant role of breakfast in attaining an optimal nutritional profile (31). The positive side of dietary pattern includes a high level of daily breakfast intake among Serbian children (84.5%), which is higher than our previous findings on Serbian adolescents (78.2%) (32). It is also slightly higher than the pooled average reports from European countries (80% of children); however, it is still lower than reports from Nordic countries, Portugal, Spain, Russian Federation, Ireland, and Montenegro (90% of children) (33–35). There is evidence that breakfast consumption is generally related to children's age, where higher breakfast skipping has been increasing as children get older, particularly in girls (36, 37). Several prospective studies support the association between breakfast consumption and a lower risk of obesity in adults (38, 39). In line with those findings, the current study also suggests an increased risk of being obese among children who were reported not to have breakfast every day [OR = 1.50 (1.05–2.14)].

The area that requires improvement relates to increasing fruit and vegetable consumption. Although global recommendation for a healthy diet suggests a daily intake of fruit and vegetables (40), the parents reported that 39.5 and 37% of Serbian children had daily fruit and vegetable consumption, respectively. The observed percentages are slightly below (42.5%) and above the average (22.6%) for daily fruit and vegetable intake, respectively, observed for 6–9-year-old children in 23 European countries (23, 33). However, our data are still far from reports that come from Nordic countries where consumption of fruit and vegetables are more than 52% (33, 35). We found a significant gender difference in vegetable consumption, with girls more frequently having vegetables more than 4 days a week. The same gender difference in consumption of vegetables was also observed in a study of Swedish 11-year-old children (35). Although we did not find any association between fruit and vegetable consumption and overweight and obesity, some studies suggested lower consumption of fruit and vegetables in overweight than normal-weight children (41).

The findings of the present study highlight the need for actions to decrease the consumption of nutrient-poor foods high in salt, sugar, and saturated fats. Our data suggest that 12.4, 20.2, and 13.4% of Serbian children consume daily savory snacks, sweets, and soft drinks, respectively, which is higher than pooled estimates observed on the European level (33). In addition, we found that children who were reported having a higher frequency of consumption of soft drinks have a higher risk of being overweight and obese. These results are consistent with studies that reported a positive association of soft drink consumption with overweight and obesity (42, 43). Interestingly, we also found a positive association between soft drink consumption and underweight. Although we did not find similar reports from the literature, unhealthy dietary behaviors could be associated with a lower frequency of consumption of nutrient-rich food (35) that could lead to some forms of malnutrition, including underweight (40).

Using a standardized WHO COSI protocol to monitor weight status and dietary patterns on the nationally representative sample is one of the present study's significant strengths. Standardized and accurate data on children's weight status and dietary habits allows comparisons within European countries and will be updated in future years. There are several limitations to be noted. The cross-sectional study design did not allow inferring a causal relation. The present study used a self-reported dietary questionnaire that had not been validated and may have limited accuracy. We did not collect information on foods' portion sizes to identify chilren's prevalence meeting specific nutrition recommendations. Our study identified a negative trend of underweight, overweight, and obesity pandemic among Serbian children compared to our previous reports (3, 24). We found some bright spots in dietary habits, such as the high prevalence of Serbian children that regularly consume breakfast and some areas that require improvements, including low fruit and vegetable and high nutrition-poor food consumption. Urgent actions are needed to ensure children consume healthy diets, including adequate quantities and appropriate proportions of fruit, vegetables (44), limiting the intake of free sugars (45), salt (46), saturated fats, and highly processed foods (47). The present findings could provide vital support for government actions to implement and evaluate effective and appropriate strategies to combat underweight and overweight.

## Data Availability Statement

The raw data supporting the conclusions of this article will be made available by the authors, without undue reservation.

## Ethics Statement

The studies involving human participants were reviewed and approved by World Health Organization (No. 2018/873491-0). Written informed consent to participate in this study was provided by the participants' legal guardian/next of kin.

## Author Contributions

PB participated in acquiring data, statistical analysis, and wrote the initial manuscript. SO contributed to the conception and design of the study, participated in statistical analyses, and reviewed and revised the manuscript. VD contributed to the conception and design of the study, coordinated and supervised data collection, and reviewed literature and critically reviewed the manuscript. LM participated in acquiring data, organized the database, contributed in statistical analysis, reviewed literature, and contribute in writing the introduction. DC participated in acquiring data and contributed in writing the methods. NT participated in acquiring data and contributed in writing the results. SH participated in acquiring data and contributed in writing the method and the results. All authors contributed to the article and approved the submitted version.

## Conflict of Interest

The authors declare that the research was conducted in the absence of any commercial or financial relationships that could be construed as a potential conflict of interest.
